# New classification‐oriented treatment strategy for portal vein thrombosis after hepatectomy

**DOI:** 10.1002/ags3.12383

**Published:** 2020-08-06

**Authors:** Shinji Onda, Kenei Furukawa, Yoshihiro Shirai, Ryoga Hamura, Takashi Horiuchi, Jungo Yasuda, Hironori Shiozaki, Takeshi Gocho, Hioaki Shiba, Toru Ikegami

**Affiliations:** ^1^ Department of Surgery The Jikei University School of Medicine Tokyo Japan

**Keywords:** anticoagulants, hepatectomy, multidetector computed tomography, portal vein, venous thrombosis

## Abstract

**Aim:**

This study sought to evaluate the incidence, risk factors, and clinical outcomes of portal vein thrombosis after hepatectomy. Furthermore, we proposed a novel classification and treatment strategy for portal vein thrombosis after hepatectomy.

**Methods:**

We retrospectively analyzed 398 patients who underwent hepatectomy and enhanced computed tomography imaging within 14 days after surgery in our hospital from 2009 to 2019. Portal vein thrombosis was classified into three categories according to the location of the thrombus – main, hilar, and peripheral – with main portal vein thrombosis further subclassified into three grades. Each patient's treatment strategy was determined based on their portal vein thrombosis classification and grading. From 2015, enhanced computed tomography imaging was performed routinely on patients who underwent anatomical hepatectomy on postoperative day 7.

**Results:**

Portal vein thrombosis was diagnosed in 57 patients (14.3%) during the study period. Multivariate analysis revealed that a Pringle maneuver time of 75 minutes or longer was a significant predictor of portal vein thrombosis (*P* = .012). In total, 52 patients (91%) with portal vein thrombosis recovered by surgery, anticoagulant therapy, or without specific treatment. There was no instance of mortality recorded.

**Conclusions:**

Patients who undergo hepatectomy are at high risk for portal vein thrombosis, especially when the Pringle maneuver time is long. The proposed classification and treatment strategy may be useful for clinical management of patients with portal vein thrombosis after hepatectomy.

## Graphical abstract

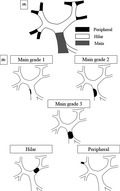

## 1 INTRODUCTION

Hepatectomy has been accepted as the standard treatment for patients with benign and primary liver cancer, as well as colorectal liver metastasis, with well‐preserved liver function.^1^ Despite recent developments in surgical techniques and perioperative management, postoperative complications may still lead to mortality. Portal vein thrombosis (PVT) is a frequent complication in cirrhosis; several factors are involved in PVT development and progression.^2^ Similarly, PVT is a potentially life‐threatening complication that also occurs after hepatobiliary surgery. PVT incidence after hepatic resection reportedly ranged from 2.1% to 9.1% in previous studies.^3^, ^4^, ^5^, ^6^ Postoperative PVT is typically asymptomatic but can cause acute intestinal ischemic necrosis, serious liver damage due to a lack of blood flow, and portal hypertension. Because there are few studies available in the literature on postoperative PVT after hepatectomy, its risk factors and appropriate management protocols are likely inadequately reported. The Yerdel et al^7^ classification of PVT was originally developed to manage mainly chronic or organized PVT in the main portal trunk in patients undergoing liver transplantation. The characteristics of patients with PVT differ from those of patients with liver transplantation. Further, various regions and degrees of PVT may lead to different clinical courses and management. As such, classifying thrombosis of branches of PVT is needed, as are novel treatment strategies based on the location of the PVT.

This study, therefore, sought to evaluate the incidence, risk factors, treatment, and clinical outcomes of postoperative PVT after hepatectomy and proposed a novel classification and management scheme for addressing PVT after hepatectomy.

## 2 MATERIALS AND METHODS

### 2.1 Patients and methods

The study included 742 consecutive patients who underwent hepatectomy for benign or malignant disease at the Jikei University Hospital from January 2009 to December 2019. Patients who underwent enhanced computed tomography (CT) imaging within 14 days after surgery were included and studied retrospectively. The ethics committee of the Jikei University School of Medicine approved the study protocol [27‐177(8062)]. Written informed consent was obtained from all included patients. Data on clinical and laboratory parameters were collected retrospectively from each patient's medical records. The indocyanine green retention test at 15 minutes and/or 99mTc‐GSA scintigraphy were routinely performed preoperatively to evaluate liver function. Meanwhile, all patients were examined preoperatively by enhanced CT and/or Gd‐EOB‐DTPA–enhanced magnetic resonance imaging.

The clinical diagnosis of PVT after hepatectomy was confirmed using enhanced CT imaging. From January 2009 to December 2013, CT was conducted using a 16‐detector‐row CT machine, while, from January 2014 to December 2019, it was performed using a 128‐detector‐row CT machine. CT was performed with a 1.5‐ to 5‐mm slice thickness. Enhanced CT was conducted on patients who presented with abdominal pain, fever, abnormal inflammation markers, liver enzyme levels, FDP levels, and D‐dimer levels. Starting from 2015, enhanced CT was performed routinely in patients who underwent anatomical hepatectomy (≥ Couinaud 1 segment) on postoperative day (POD) 7.

All patients were tested routinely to discern the complete blood count, liver and kidney function, C‐reactive protein level, and blood coagulation parameters, including PT activity, PT‐INR, APTT, and fibrinogen level on preoperative day 1‐3, PODs 1, 3, 5, and 7. Since 2015, serum FDP level, D‐dimer level, and AT‐III activity were routinely measured on preoperative day 1‐3, PODs 1, 3, 5, and 7. We did not routinely use prophylactic anticoagulants postoperatively. Postoperative variables included laboratory data, duration of hospital stay, reoperation for postoperative bleeding, 30‐day mortality, and in‐hospital mortality. The laboratory tests performed during the postoperative period were the same ones performed in the preoperative period. Postoperative bile leakage was defined as grade B/C according to the International Study Group of Liver Surgery.^8^ Clinical characteristics were compared between patients with and without PVT from 2009 to 2019. The results of serum coagulation tests, which assessed PT activity, APTT, fibrinogen level, FDP level, D‐dimer level, and AT‐III level on preoperative day 1‐3 and POD 1, were compared between patients with and without PVT from 2009 to 2019.

### 2.2 New classification of PVT

**FIGURE 1:**
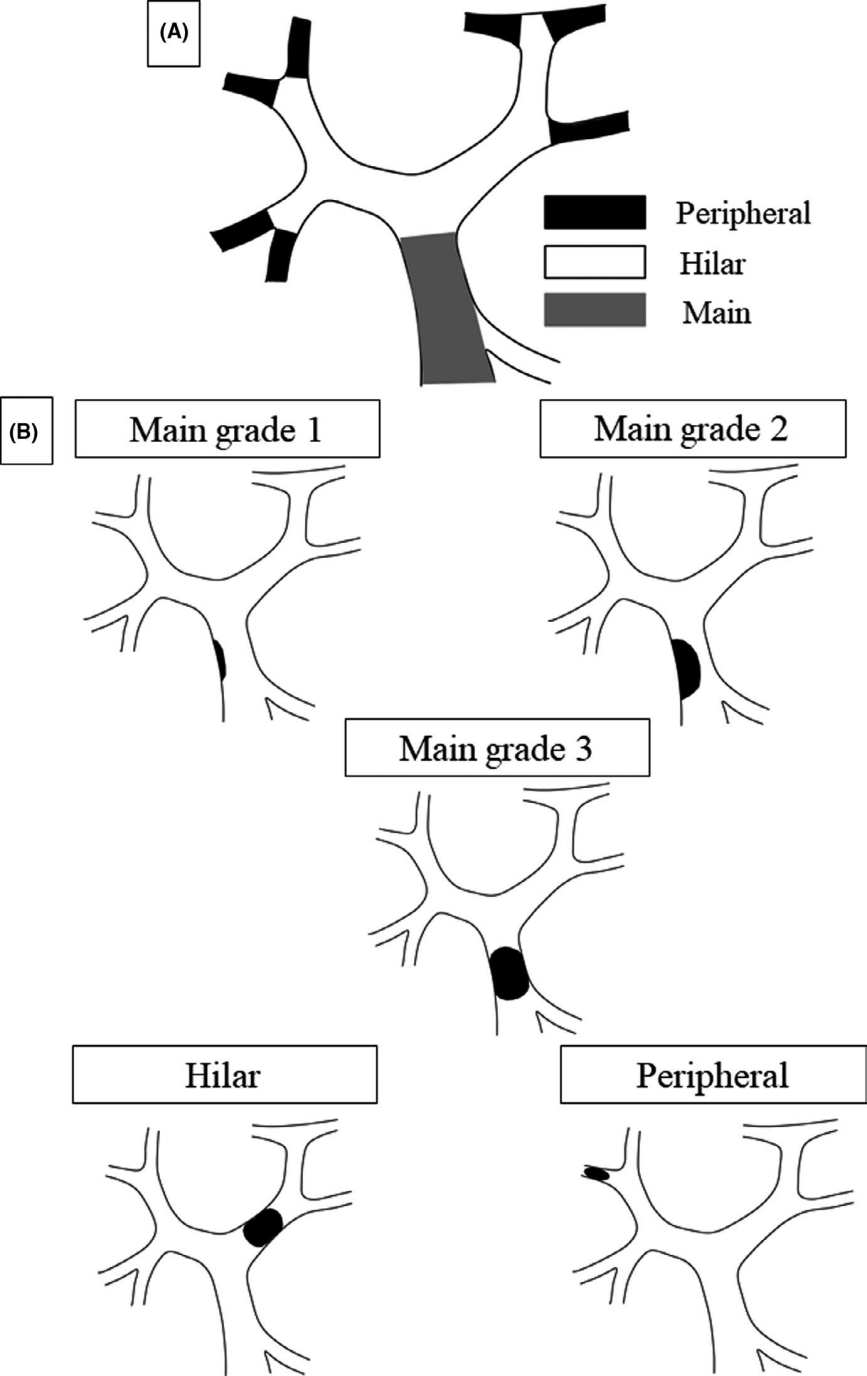
The proposed classification of PVT according to location (A) and grading (B)

As part of this research, we propose a novel classification system for PVT after hepatectomy. PVT, in this study, was classified into the following three categories according to the site of thrombus (Figure 1).

1. Main: thrombus is present only in the main portal vein (MPV) or in the MPV and superior mesenteric vein. Main PVT was further classified into the following three grades.
  - Grade 1: minimal or luminal MPV thrombosis (<50% luminal space).
  - Grade 2: partial MPV thrombosis (>50% luminal space).
  - Grade 3: complete or near obstruction of the MPV
2. Hilar: thrombus is present in the major branches, including the first branches, anterior branch, posterior branch, and umbilical portion
3. Peripheral: thrombus is present in subsegmental branches (Couinaud's segments 1‐8).

### 2.3 Treatment strategy for PVT

For patients with PVT, the treatment strategy selected, according to the PVT grade, was as follows:

1. Peripheral and main grade 1: observation
2. Main grade 2 and hilar: anticoagulation therapy
3. Main grade 3: surgical thrombectomy

Patients with peripheral PVT only (no main grade 1) underwent anticoagulation therapy when the thrombus was located in the root of the subsegment branches, while patients with minimal hilar thrombosis did not undergo anticoagulation therapy.

We performed anticoagulation therapy using intravenous, unfractionated heparin for about 7 days, followed by warfarin or direct oral factor Xa inhibition for 3 months. AT‐III concentrate was administered for more than 3 days when the serum AT‐III activity was lower than 70%. Because anticoagulant treatment guidelines of PVT are not established, anticoagulation therapy did not involve specific anticoagulants, and various anticoagulants were irregularly given. We did not introduce anticoagulation therapy in patients who were at a high risk of bleeding. The target APTT was 1.5 times the normal value during heparin therapy, and the target PT‐INR was 1.5 to 2.5 during warfarin therapy. Follow‐up with enhanced CT took place after within 14 days; then the patients underwent enhanced CT imaging or enhanced magnetic resonance imaging within 6 months after the PVT diagnosis. Resolved thrombus was defined as the complete or near complete resolution of the thrombus on imaging findings within 14 days or 6 months after the PVT diagnosis, while organized thrombus was defined as significant stenosis or occlusion of the vessels due to thrombosis on imaging findings at least 1 month after the PVT diagnosis.

### 2.4 Surgical procedures

The extent of liver resection was based on the patient's functional liver reserve and the volumetric CT analysis results. The Pringle maneuver was performed as necessary by tightening a rubber tube around the entire hepatoduodenal ligament (total Pringle maneuver) or selectively around the left of right portal pedicle using an extra Glissonean approach (selective Pringle maneuver). Cycles of 15 minutes of clamping were alternated with 5 minutes of reperfusion. The portal vein flow was assessed by Doppler ultrasonography during reperfusion time. The hepatic parenchyma was divided, using a Cavitron ultrasonic surgical aspirator or the crush‐clamping method, and each vessel encountered was ligated or clipped. For anatomical segmentectomy and subsegmentectomy, Glissonean pedicle transection or ultrasound‐guided dye injection was performed as described previously.^9^, ^10^ The intrahepatic artery, portal vein, and hepatic vein flow were routinely assessed by Doppler ultrasonography after hepatic resection.

### 2.5 Statistical analysis

Continuous variables are presented as means ± standard deviations or medians with ranges, while categorical variables are presented as frequencies and percentages. Univariate analysis was performed using Fisher's exact test or Welch's *t* test, as appropriate. Multivariate analysis was performed to identify significant predictive variables. Receiver operating characteristic (ROC) curve analysis was performed for correlation analysis. The results were considered statistically significant when *P*‐values were lower than .05. Statistical analyses were performed using SPSS version 20 software program (IBM Corp).

## 3 RESULTS

This study included 398 patients aged 18‐90 years (mean age: 63.6 years); 281 patients (71%) were men and 117 (29%) were women. Laparoscopic surgery was performed on 33 patients (8%), while 268 underwent anatomical hepatectomy, and 130 underwent partial hepatectomy. The overall mean operation time was 465 minutes, and the mean estimated blood loss volume was 1174 mL. The intraoperative red blood cell transfusion rate was 29%. Due to postoperative bleeding, six patients (1.5%), who were all non‐PVT patients, underwent reoperation. The median duration of postoperative hospital stay was 13 days. The overall 30‐day mortality rate was 0.5% and the postoperative in‐hospital mortality rate was 1.8%.

Among the 398 patients considered, 57 (14.3%) were diagnosed with PVT. The comparison of clinical data between patients with and without PVT from 2009 to 2019 is shown in Table 1. Subsegmentectomy (*P* = .033), operation time (*P* = .014), and Pringle maneuver time (*P* < .001) were significantly higher in patients with PVT than in patients without PVT, while partial hepatectomy (*P* = .003) was significantly lower among patients with PVT than those without PVT. ROC curve analysis revealed cutoff values of 430 and 75 minutes for the operation time and Pringle maneuver time, respectively. Multivariate analysis revealed that a Pringle maneuver time of 75 minutes or longer (odds ratio: 2.72, 95% confidence interval: 1.25‐5.92; *P* = .012) is a significant predictor of PVT (Table 2). A comparison of the coagulation tests performed on preoperative day 1‐3 and POD 1 between patients with and without PVT between 2009 and 2019 is shown in Table 3. The serum PT activity of patients with PVT on POD 1 was significantly lower than that of patients without PVT (*P* = .004). According to the ROC curve analysis, a cutoff value of 61.5% for serum PT showed sensitivity of 42.6% and specificity of 80.7% (AUC = 0.618, *P* = .004).

**TABLE 1:** Clinicopathological characteristics of patients with PVT after hepatectomy

	No PVT (n = 341)	PVT (n = 57)	*P*‐value
Age (y)	63.2 ± 13.3 (18‐90)	65.5 ± 8.7 (38‐83)	.214
Sex, male	240 (70%)	41 (72%)	.876
Diagnosis			
HCC	157 (46%)	34 (60%)	.063
ICC	18 (5%)	1 (2%)	.497
Hilar cholangiocarcinoma	9 (3%)	0 (0%)	.369
Metastatic	101 (30%)	15 (26%)	.753
Other malignant	16 (5%)	2 (4%)	1.000
Living liver donor	15 (4%)	1 (2%)	.487
Other benign	25 (7%)	4 (7%)	1.000
Patient's comorbidities			
Hypertension	136 (40%)	20 (35%)	.559
Cardiovascular disease	30 (9%)	5 (9%)	1.000
Cerebrovascular disease	15 (4%)	1 (2%)	.487
Preoperative antithrombotic medication	46 (13%)	6 (11%)	.673
Preoperative hemoglobin <12.0 g/dL	89 (26%)	14 (25%)	.872
Preoperative platelet count <100 × 10^3^/µL	26 (8%)	3 (5%)	.783
Preoperative albumin <3.6 g/dL	73 (21%)	8 (14%)	.285
Preoperative CRP >0.3 mg/dL	92 (27%)	13 (23%)	.627
Preoperative PT activity <70%	17 (5%)	1 (2%)	.490
BMI ≥25 kg/m^2^	94 (28%)	19 (33%)	.428
ICG‐R15 >10%, n = 375	190 (56%)	39 (68%)	.134
Child‐Pugh class A:B	326:15	56:1	.487
Surgical procedure			
Partial resection	120 (35%)	10 (18%)	.009
Subsegmentectomy	34 (10%)	13 (23%)	.013
Segmentectomy	59 (17%)	14 (25%)	.198
Bisegmentectomy	122 (36%)	20 (35%)	1.000
Trisegmentectomy	6 (2%)	0 (0%)	.600
Laparoscopic surgery	31 (9%)	2 (4%)	.200
Operation time (min)	456 ± 174	520 ± 206	.014
Blood loss (mL)	1142 ± 2256	1366 ± 3517	.527
Intraoperative RBC transfusion	98 (29%)	16 (28%)	1.000
Pringle's maneuver	276 (81%)	49 (86%)	.461
Total:Selective	201:75	29:20	.061
Pringle time (min)	88 ± 54	128 ± 77	<.001
Resected liver weight (g)	383 ± 515	430 ± 348	.509
Postoperative bile leakage grade B/C	27 (8%)	9 (16%)	.076
Histological liver cirrhosis, F4 stage	40 (12%)	7 (12%)	.828

Note

Continuous variables were expressed as mean ± SD.

Abbreviations: BMI, body mass index; HCC, hepatocellular carcinoma; ICC, intrahepatic cholangiocarcinoma; ICG‐R15, indocyanine green retention test after 15 min; PVT, portal vein thrombosis.

**TABLE 2:** Multivariate analysis to identify risk factors for PVT after hepatectomy

	Odds ratio	95% CI	*P*‐value
Subsegmentectomy	0.51	0.23‐1.11	.088
Operation time ≥430 min	1.27	0.63‐2.57	.499
Pringle time ≥75 min	2.72	1.25‐5.92	.012

Abbreviation: CI, confidence interval.

**TABLE 3:** Comparison of the results of coagulation tests performed on preoperative day 1‐3 and postoperative day 1 between patients with PVT and patients without PVT after hepatectomy between 2009 and 2019

	No PVT	PVT	*P*‐value
Preoperative			
PT activity (%), n = 394	90.4 ± 11.4	89.0 ± 10.2	.415
APTT (s), n = 387	30.3 ± 5.56	29.0 ± 2.74	.082
Fibrinogen (mg/dL), n = 367	348 ± 114	345 ± 117	.870
FDP (µg/mL), n = 123	4.2 ± 6.0	3.0 ± 1.2	.220
D‐dimer (µg/mL), n = 126	1.7 ± 2.7	2.0 ± 3.3	.890
AT‐III activity (%), n = 126	95.5 ± 18.1	97.0 ± 29.1	.690
Postoperative day 1			
PT activity (%), n = 395	59.3 ± 14.5	53.5 ± 12.4	.004
APTT (s), n = 394	30.1 ± 3.91	29.6 ± 3.30	.388
Fibrinogen (mg/dL), n = 388	281 ± 91.0	259 ± 79.4	.089
FDP (µg/mL), n = 232	20.1 ± 28.8	21.9 ± 13.6	.683
D‐dimer (µg/mL), n = 233	9.70 ± 8.82	10.6 ± 6.32	.534
AT‐III activity (%), n = 255	64.7 ± 18.8	62.6 ± 14.8	.502

Note

Values were expressed with mean ± SD.

Abbreviations: APTT, activated partial thromboplastin time; AT, antithrombin; FDP, fibrin degradation product; PT, prothrombin time; PVT, portal vein thrombosis.

Among the PVT patients, main thrombosis was found in 14 patients (25%), hilar thrombosis was found in 30 patients (53%), and peripheral thrombosis was found in 13 patients (23%). Of the patients with main thrombosis, grade 1 was noted in eight patients (14%), grade 2 was noted in four patients (7%), and grade 3 was noted in two patients (3%) (Table 4). Of the patients with main thrombosis, three had both main and hilar thrombosis.

**TABLE 4:** The proposed classification and grading of PVT and the treatment outcome

Classification and grading	Number n = 57	Treatment		Outcome
		Anticoagulation	Thrombectomy	Resolved
Main	14 (25%)	7 (50%)	2 (14%)	13 (93%)
Grade 1	8 (14%)	1 (13%)	0 (0%)	8 (100%)
Grade 2	4 (7%)	4 (100%)	0 (0%)	3 (75%)
Grade 3	2 (3%)	2 (100%)	2 (100%)	2 (100%)
Hilar	30 (53%)	24 (80%)	0 (0%)	24 (80%)
Peripheral	13 (23%)	5 (38%)	0 (0%)	12 (92%)

Abbreviation: PVT, portal vein thrombosis.

Out of the 57 patients with PVT, 24 patients (42%) were asymptomatic. PVT diagnosis was confirmed at a mean of six (range: 1‐11) days postoperatively. In the PVT patients, the median duration of the postoperative hospital stay was 17 days, which was significantly longer than that of 13 days found among patients without PVT (*P* = .011).

### 3.1 Treatments and outcomes

**FIGURE 2:**
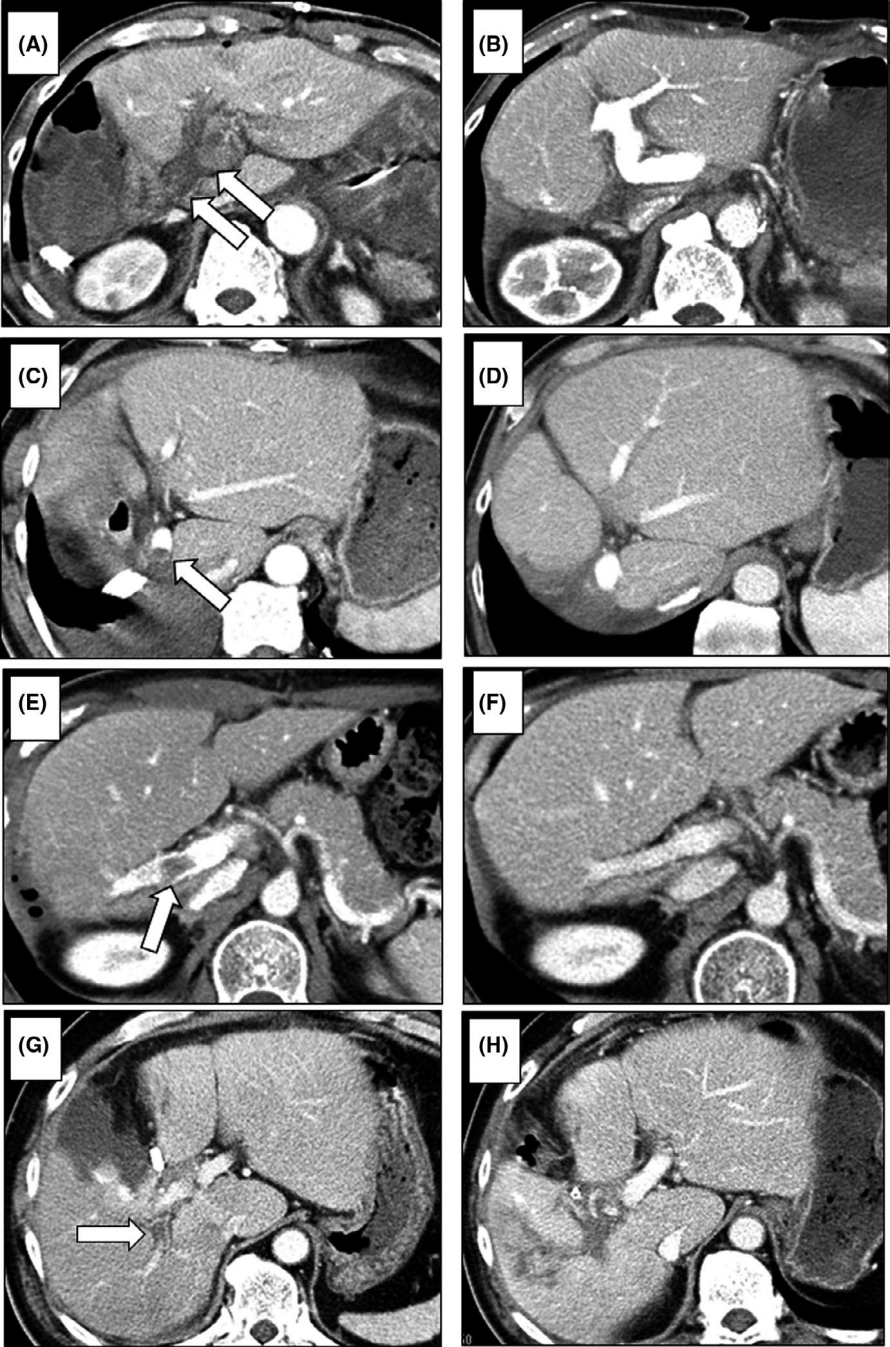
CT findings of PVT. In patients with complete obstruction of MPV thrombosis—main grade 3 (CT on POD 1) (arrows) (A)—the thrombus was removed by urgent surgical thrombectomy (CT on POD 21) (B). In patients with a small MPV thrombosis—main grade 1 (CT on POD 9) (arrow) (C)—the thrombus resolved without treatment (CT at 6 mo after surgery) (D). In patients with right‐branch PVT—hilar thrombosis (CT on POD 7) (arrow) (E)—the thrombus resolved with anticoagulation therapy (CT at 3 mo after surgery) (F). In patients with posterior branch PVT—hilar thrombosis (CT on POD 10) (arrow) (G)—the thrombus organized in spite of anticoagulation therapy (CT at 1 mo after surgery) (H)

Of the 57 patients with PVT, 34 patients (five patients with main thrombosis, 24 patients with hilar thrombosis, and five patients with peripheral thrombosis) received anticoagulant therapy alone, and the thrombus resolved in 29 (85%) patients (four patients with main thrombosis, 21 patients with hilar thrombosis, and four patients with peripheral thrombosis) and became organized in five patients (Table 5, Figure 2). Of the 21 patients who did not receive anticoagulant therapy, the thrombus resolved in all cases. Two patients with main grade 3 thrombosis successfully underwent urgent surgical thrombectomy on POD 1. Therefore, 52 patients (91%) with PVT recovered by surgery, anticoagulant therapy, or without specific treatment. The PVT location and its treatment outcome are shown in Table 4. The complete clearance rate of PVT was 93% in the MPV, 80% in the hilar region, and 92% in the peripheral region, respectively. The treatment modalities and clinical outcomes are shown in Table 5.

**TABLE 5:** The anticoagulant therapy and clinical outcomes of patients with PVT except for patients who underwent thrombectomy

Anticoagulant therapy	Number n = 55	Outcomes	
		Resolved	Organized
Anticoagulant therapy	34	29	5
IUH	2	1	1
IUH and AT‐III concentrate	1	0	1
IUH followed by warfarin	16	14	2
IUH followed by Xa inhibitor	8	8	0
Warfarin alone	1	1	0
AT‐III concentrate	2	1	1
Xa inhibitor	3	3	0
LMWH	1	1	0
None	21	21	0

Abbreviations: AT, antithrombin; IUH, intravenous unfractionated heparin; LMWH, low molecular weight heparin; PVT, portal vein thrombosis.

There was no instance of postoperative liver failure or in‐hospital mortality among the patients with PVT in this study. Also, there was no case of postoperative bleeding that occurred among the patients treated with anticoagulant therapy.

### 3.2 Correlation of the PVT location and operative procedures

Regarding the PVT location, main thrombosis showed a high rate of incidence in the right or left hemihepatectomy, while hilar thrombosis showed a high rate of incidence in anterior or posterior segmentectomy and left lateral or medial segmentectomy and subsegmentectomy (Table 6).

**TABLE 6:** Correlation of the PVT location and operative procedures

Operative procedures	Number n = 57	Location of PVT		
		Main	Hilar	Peripheral
Rt/Lt hemi‐hepatectomy	17 (30%)	11 (65%)	3 (18%)	3 (18%)
Central bisegmentectomy	3 (5%)	0	1 (33%)	2 (67%)
Ant/Post segmentectomy	8 (14%)	0	7 (88%)	1 (12%)
Left lateral/medial segmentectomy	6 (11%)	0	6 (100%)	0
Subsegmentectomy	13 (23%)	1 (8%)	9 (69%)	3 (23%)
Partial hepatectomy	10 (17%)	2 (20%)	4 (40%)	4 (40%)

Abbreviation: PVT, portal vein thrombosis.

## 4 DISCUSSION

We found that the incidence of PVT after hepatectomy was 14.3%, and a duration of the Pringle maneuver of 75 minutes or longer is a significant predictive factor of PVT after hepatectomy. Furthermore, we proposed a novel classification system and treatment strategy for PVT after hepatectomy, which is easy to use clinically and provides a satisfactory outcome with a high rate of complete clearance of thrombosis.

The pathogenesis of thrombosis is based on Virchow's triad – venous stasis, hypercoagulable state, and endothelial injury^11^ – and PVT is provoked by these three factors, which are interdependent and often coexist with one another.^12^ A hypercoagulable state after hepatectomy has been reported as seen in this study, serum PT activity on POD 1 was lower in the PVT group than that in the control group.^13^ The Pringle maneuver is a useful approach by which to control bleeding from the portal vein during hepatectomy,^14^ but it can lead to stasis in the portal vein and endothelial injury.^12^ In this study, a longer duration of the Pringle maneuver is a significant risk factor for PVT, which is consistent with previous reports.^3^

Portal vein thrombosis can occur at any location, although it usually originates from surgically manipulated regions, such as the portal vein stump, and can be located only in the MPV due to kinking or compression. Therefore, we believe that developing a novel classification scheme for PVT after hepatectomy is necessary.

In the present study, anticoagulant therapy was introduced immediately after PVT diagnosis, where appropriate, because of the possibility of thrombosis extension and decreased portal venous flow, which may result in liver failure or portal hypertension. In our treatment strategy for PVT after hepatectomy, the patients with main grade 2 or hilar thrombosis received anticoagulation therapy, and 75% and 80% of these individuals recovered from PVT, respectively. On the other hand, most of the patients with main grade 1 or patients with peripheral thrombosis did not receive anticoagulation therapy, and PVT resolved spontaneously in all these individuals, which is consistent with the findings of a previous study.^15^ Of 30 patients with hilar thrombosis, six did not receive anticoagulation therapy because of a small thrombosis or refused treatment, yet the thrombosis resolved in all these individuals. This result suggests that small thrombi of the hilar region may be followed up for a short period without further action and, if seen to be worsening, anticoagulation therapy should be started immediately.

Incidentally, the location and etiology of thrombosis vary depending on the surgical procedure, which is supported by the result in Table 6. During hemi‐hepatectomy, a thrombus is likely to be formed in the MPV; especially in right‐sided hemi‐hepatectomy, PVT is considered to be caused by the kinking of the PV trunk or turbulent flow in the PV, as reported previously.^3^, ^5^ During segmentectomy, a thrombus is likely to be formed in the hilar region, mostly in the stumps of the vessels. As reported by Mori et al,^15^ gentle stretching and careful dissection around the PV may be necessary to prevent PVT. On the other hand, a thrombus located far away from the surgically manipulated region is considered to have formed in the MPV and, thereafter, flowed into hilar or peripheral region.

When PVT is detected in the early period after hepatectomy, the treatment effectiveness is likely to be higher than when it is detected later on after hepatectomy,^5^ which is considered to be the result of thrombus formation being fresher in the early period after hepatectomy. The occurrence of peripheral PVT or small thrombi in the MPV is less likely to provoke liver damage or lead to portal vein insufficiency. In patients with a thrombus in the root of the subsegmental branches of the PVT, anticoagulation therapy was performed to prevent expansion of the thrombus into the hilar region. In contrast, complete or near‐complete obstruction of the MPV caused by PVT – namely, main grade 3 – is an indication for urgent surgical thrombectomy. In this study, two patients in whom the thrombi were fresh and easily removed from the vascular wall underwent urgent surgical thrombectomy on POD 1. In contrast, Kuboki et al^5^ reported that it was difficult to remove a thrombus by surgical thrombectomy in patients with PVT detected on POD 6 or later because the thrombus had organized and adhered rigidly to the vascular wall by this point in time. Therefore, urgent operative thrombectomy is strongly recommended in patients with complete obstruction of the MPV caused by PVT.

Because patients with PVT do not present specific symptoms, early PVT diagnosis after hepatectomy is usually difficult. In this study, 42% of patients with PVT were asymptomatic. There is an increased chance of finding asymptomatic PVT during routine enhanced CT. However, it is unknown, in such cases, when the thrombosis started to develop, and the results of this study and other previous investigations suggest that it is reasonable to screen patients after hepatectomy on around POD 7.^3^, ^4^

Regarding diagnostic modalities for screening for PVT after hepatectomy, Doppler ultrasonography and enhanced CT are usually used. Color Doppler's sensitivity for PVT detection after hepatectomy is reported to be 56%.^5^ Ultrasonography after surgery is noninvasive but shows difficulty in detecting PVT because of the abdominal incision and increased amount of bowel gas after surgery. In contrast, the sensitivity of enhanced CT for PVT detection after hepatectomy was 100%.^5^ In addition, Yoshiya et al^3^ reported that PVT after hepatectomy is closely related with the delayed recovery of liver function and delayed liver regeneration. Therefore, making an accurate diagnosis using routine enhanced CT in the early period after hepatectomy is mandatory, and a rapid start of treatment is important.

The incidence of PVT after hepatectomy in this study was higher than that of previous studies. The higher incidence may be due to the selected patient study population because routine CT examination was performed in patients who underwent anatomical hepatectomy while excluding patients who underwent partial hepatectomy who may show a higher incidence of PVT. Because we focused on PVT during routine CT examinations, minimal or peripheral PVT were detected, and that may have been underestimated in other studies.

This study has several limitations. First, it was retrospective in nature and included patients who underwent enhanced CT postoperatively, while patients who did not undergo enhanced CT were excluded; thus, it has the potential of selection bias. Second, anticoagulation therapy did not involve specific anticoagulants, and various anticoagulants were irregularly given in this study, as anticoagulant treatment guidelines of PVT are not established. In the future, further studies are needed to establish the standard anticoagulant treatment agents for PVT. In addition, it is important to consider how to prevent postoperative PVT. Recently, an interesting study was reported that massage of hepatoduodenal ligament recovers portal vein flow immediately after the Pringle maneuver in hepatectomy.^16^ In the future, we plan to study the improvement of the Pringle maneuver technique to prevent postoperative PVT.

In conclusion, patients who undergo hepatectomy are at high risk for PVT, especially when the duration of the Pringle maneuver is long. As many patients with PVT are asymptomatic and possibly progressive during a short period, routine CT screening is necessary in the early period after hepatectomy. The proposed classification and treatment strategy, based on the location and severity of the PVT, can help to improve clinical outcomes.

## DISCLOSURE

Conflict of Interest: The authors declare no conflict of interest for this article.

Author Contribution: Conception and design of the study, analysis and interpretation of data, collection and assembly of data, drafting of the article: Shinji Onda. Analysis and interpretation of data, collection of data: Kenei Furukawa, Yoshihiro Shirai. Collection of data: Ryoga Hamura, Takashi Horiuchi, Jungo Yasuda, Hironori Shiozaki, Takeshi Gocho, Hioaki Shiba. Critical revision of the article: Toru Ikegami.
